# Mode-of-Action of Antimicrobial Peptides: Membrane Disruption vs. Intracellular Mechanisms

**DOI:** 10.3389/fmedt.2020.610997

**Published:** 2020-12-11

**Authors:** Aurélie H. Benfield, Sónia Troeira Henriques

**Affiliations:** School of Biomedical Sciences, Institute of Health & Biomedical Innovation, and Translational Research Institute, Australian Research Council Centre of Excellence for Innovations in Peptide and Protein Science, Queensland University of Technology, Brisbane, QLD, Australia

**Keywords:** peptide-lipid interactions, bacterial membrane, cellular uptake, biophysical methodologies, peptide therapeutics

## Abstract

Antimicrobial peptides are an attractive alternative to traditional antibiotics, due to their physicochemical properties, activity toward a broad spectrum of bacteria, and mode-of-actions distinct from those used by current antibiotics. In general, antimicrobial peptides kill bacteria by either disrupting their membrane, or by entering inside bacterial cells to interact with intracellular components. Characterization of their mode-of-action is essential to improve their activity, avoid resistance in bacterial pathogens, and accelerate their use as therapeutics. Here we review experimental biophysical tools that can be employed with model membranes and bacterial cells to characterize the mode-of-action of antimicrobial peptides.

## Introduction

Antibiotic resistant bacteria are rapidly emerging while the development of new antimicrobial agents is decelerating (1–3). To fight infections caused by resistant bacteria, it is essential to develop new compounds. Antimicrobial peptides (AMPs) have attracted attention as potential alternative antimicrobial agents, as these small biological molecules kill bacteria using a mode-of-action (MOA) distinct from those used by traditional antibiotics (4).

AMPs are produced in almost all species (5), kill a broad spectrum of bacteria, fungi, protozoa and viruses, have anticancer properties (6, 7), and can kill antibiotic-resistant bacteria (8, 9). In general, AMPs are positively charged amphipathic molecules able to selectively target bacteria and kill them using two broad MOAs. In the first mechanism AMPs induce membrane disruption, leading to cell lysis and death. In the second MOA, AMPs enter cells without membrane disruption and inhibit essential intracellular functions by binding to nucleic acids or intracellular proteins (10–12).

Peptide-based antimicrobials, such as Tyrothricin, Gramicidin S (13), Vancomycin and Telavancin (14), are used in the clinic as therapeutics. Nevertheless, the widespread application of AMPs is limited by a perception that peptides are expensive to produce, susceptible to proteases, and display high cytotoxicity (15–17). Peptide production costs have decreased over the past years due to advances in solid- and liquid-phase peptide synthesis (18, 19), and production of recombinant peptides in *Escherichia coli* (20) and yeast (21). Peptides can be engineered to increase their chemical and proteolytic stability via backbone cyclization (22), side chain-to-side chain cyclization (23), or the inclusion of stereochemical amino acids (24). Furthermore, toxicity to the host can be reduced, and potency can be improved, if we understand their MOA (16, 25–28).

In this mini-review, we highlight some experimental biophysical techniques that can be employed to investigate the complex MOA of AMPs. Identifying whether particular AMPs act by disrupting bacterial membranes, or by interfering with an intracellular pathway, is key to rationally improve efficacy, stability and safety of AMPs, and develop novel antimicrobial therapeutics.

## Characterization of Peptide-Lipid Binding Using Model Membranes

AMPs generally target and bind bacterial membranes via peptide-lipid interactions. Model membrane systems, including Langmuir monolayers, liposomes, and solid supported bilayers, have been used to screen peptide-membrane interactions and investigate the effect of peptides on the structure of lipid bilayers. Although they are less complex than bacterial membranes, model membranes are useful to investigate individual membrane components (29, 30). They can be produced with defined lipid compositions, biophysical properties, and conditions (e.g., size, charge, pH, ionic strength); thereby reducing the variables present in biological assays.

Liposomes are particularly useful as a model membrane: they are versatile, easy to prepare, and can be used in several methodologies. Liposomes can be prepared with synthetic lipids present in bacterial membranes, such as phospholipids with phosphatidylglycerol-, or phosphatidylethanolamine-headgroups and cardiolipin (31) or can be prepared with lipids extracted directly from bacterial membranes. Liposomes are unilamellar or multilamellar structures obtained by suspending lipids in an aqueous solution and by sonicating, or extruding them through a membrane filter with a defined pore size, such as 50 nm, 100 nm and 1 μm, to prepare small, large and giant vesicles, respectively.

**Figure 1:**
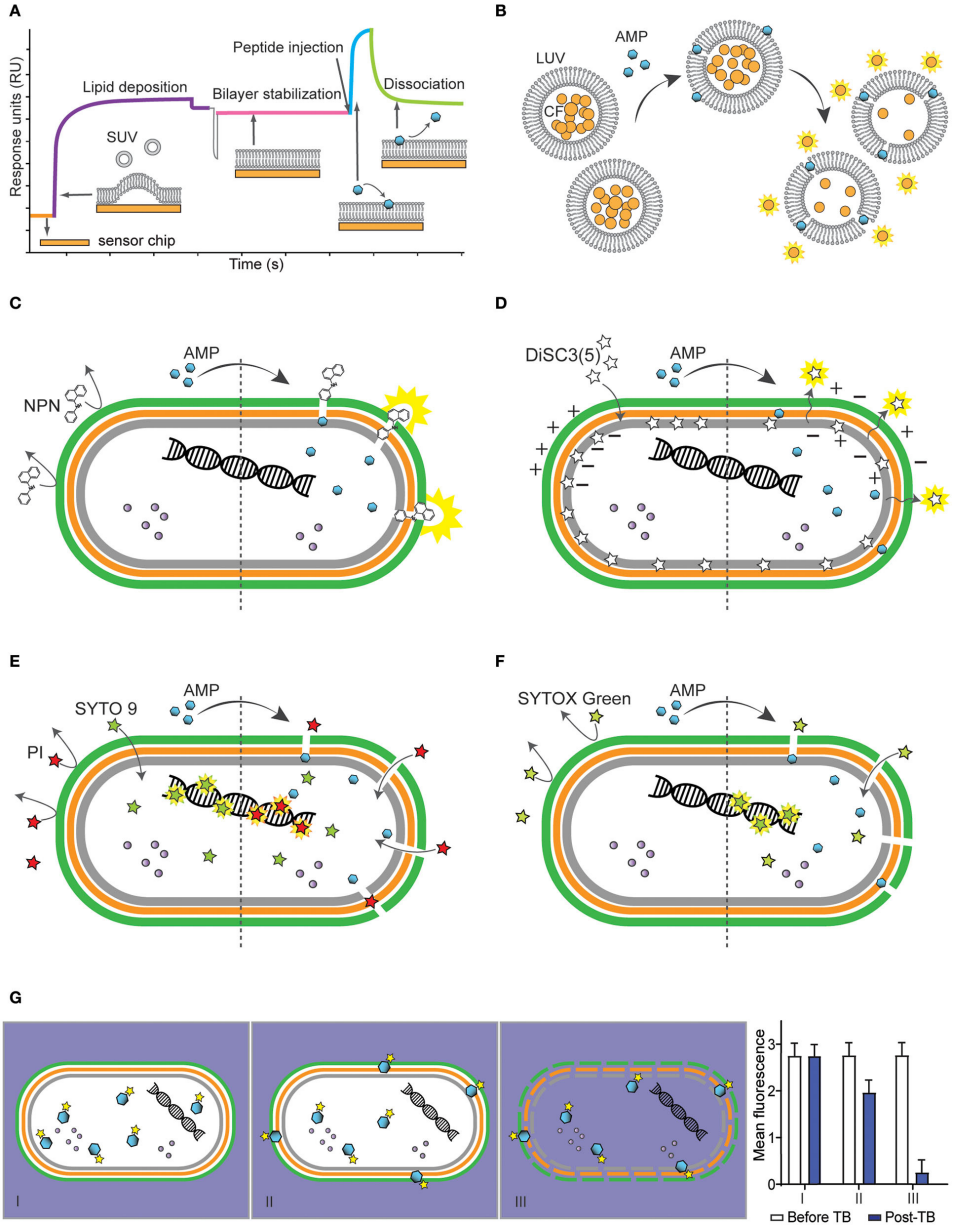
Methodologies to determine interactions of antimicrobial peptides (AMPs) with bacterial membranes using model membranes **(A,B)** and bacterial cells **(C-G)**. **(A)** Schematic representation of a sensorgram obtained with surface plasmon resonance (SPR) to monitor peptide-lipid interactions. The response units (RU) increase when small unilamellar vesicles (SUV) are injected to cover the chip (orange) and form a bilayer (purple). Once the bilayer is stabilized (pink), AMPs are injected and association with the lipid bilayer (blue) is monitored in real time. The stronger the binding of the antimicrobial peptide (AMP) to the lipid system, the higher the response units. When the peptide injection stops, it is possible to monitor the dissociation of the peptides from the lipid bilayer (green). **(B)** Illustration of the leakage assay using large unilamellar vesicles (LUV) filled with carboxyfluorescein (CF), whose fluorescence is self-quenched when packed inside LUVs at high concentration. In the presence of permeabilizing AMPs, CF escapes into the aqueous environment and becomes fluorescent. **(C–F)** Schematic of a Gram-negative bacterium with inner membrane (gray), peptidoglycan layer (orange), outer membrane (green) and intracellular content such as proteins (purple spheres) and nucleic acids (helix) and the effect of fluorescent dyes before (left) and after (right) treatment with a membrane-active AMP. **(C)** Illustration of *N*-phenyl-1-napthylamine (NPN) becoming highly fluorescent when in hydrophobic environment such as lipid membranes, subsequently damaged by AMPs. **(D)** Illustration of 3,3′-Dipropylthiadicarbocyanine iodide [DiSC_3_(5)] packed within inner membrane of bacterium. DiSC3(5) is released into the aqueous environment and becomes highly fluorescent (right) once membrane is depolarized by binding of AMPs. **(E)** Illustration of bacterial cells incubated with propidium iodide (PI) and SYTO 9, to label lysed/dead and viable cells, respectively. Left side shows PI unable to go through intact membrane, unlike SYTO 9 that can penetrate intact membrane and bind to DNA. Once AMP damages membrane, PI enters the cell and displaces SYTO 9 (right). **(F)** Illustration of SYTOX Green which is highly fluorescent if bound to DNA, however, unable to go through bacterial membrane unless membrane is damaged by the AMP. **(G)** Schematic of internalization of bacterial cell by fluorescently labeled AMP and graph showing fluorescence measurement before and after addition of trypan blue (TB). Panel I shows an AMP able to enter bacteria cells without damaging membrane, panel II shows an AMP entering cells with a portion located on the membrane, and panel III shows internalization of an AMP and permeabilization of bacterial membrane resulting in drop of fluorescence readings after addition of TB quenching fluorescence of labeled AMP.

Surface plasmon resonance (SPR) (Figure 1A) can be used to study peptide-lipid binding affinity in real-time and without requiring fluorescently labeled peptide. In this assay, liposomes are deposited onto the surface of a sensor chip covered with polydextran (e.g., L1 Sensor Chip from GE Biacore systems) to form a stable lipid bilayer (32). Peptide solution is injected over the lipid bilayer and peptide-lipid binding is monitored via variation of refractive index over time. Sensorgrams can be used to calculate peptide-lipid binding association (k_on_), dissociation (k_off_) rate constants, equilibrium dissociation constant (K_D_) and membrane partition coefficients (K_p_) (32, 33). It is possible to predict cytotoxic properties of AMPs by comparing their peptide-lipid binding affinity. AMPs with high binding affinity for negatively charged membranes and with weak affinity for zwitterionic membranes are normally selective toward bacteria and not toxic to host cells (34).

Many AMPs kill bacteria by inducing membrane disruption and leakage of bacterial content. Leakage assays with model membranes (Figure 1B) can be used to investigate the ability of AMPs to disrupt lipid bilayers. In these assays, an aqueous soluble fluorescent dye, such as carboxyfluorescein (35–37) or calcein (38, 39), is entrapped into large unilamellar vesicles at self-quenching concentrations. Dye-loaded vesicles are resuspended in buffer by gel filtration and their lipid concentration quantified using the Stewart assay (40). If peptides permeabilize vesicles, the fluorescent dye is released into solution, resulting in increased fluorescence emission signal. This assay can be performed in a 96-well plate format to reduce the volume of reagents and peptides (41). It can also be used to investigate membrane selectivity in a competitive lipid environment (42). For example, AMPs can be incubated with a mixture of liposomes of distinct composition to quantify selective disruption of negatively charged over neutral liposomes. Although leakage assays do not provide information about the disruption mechanism involved (e.g., toroidal pore, barrel pore, or carpet mechanism), they inform on whether AMPs can disrupt lipid bilayers, can be used to investigate membrane selectivity (42).

Molecular dynamic simulations using atomistic or coarse-grained models of lipid bilayers, can also be used to characterize peptide-lipid interactions and to gain information on the disruption mechanism used by specific AMPs (43–46). More complex bacterial cell wall models (e.g., peptidoglycan network, outer membrane of Gram-negative bacteria) (47–49) have been developed and can be used to simulate interactions of peptides with bacterial cell walls, and predict membrane disruption properties (50).

## Examining Integrity and Function of Bacterial Membranes Using Membrane Dyes

Membrane dyes and fluorescence spectrophotometer plate readers can be used with bacteria cells to study the effect of AMPs on the integrity of specific layers of bacterial membranes. For instance, the fluorescence of the lipophilic dye *N*-phenyl-1-napthylamine (NPN) can inform on the ability of AMPs to permeabilize the outer membrane of Gram-negative bacteria (Figure 1C). NPN emits weak fluorescence in aqueous environment and is highly fluorescent in hydrophobic environment found in lipid membranes. NPN cannot insert into intact bacteria membranes; however, when AMPs disturb the outer membrane of Gram-negative bacteria, NPN gains access to lipid layers in the outer membrane and/or in the cytoplasmic membrane (51–53) and its fluorescence emission intensity increases. This assay can be done using a 96-well plate format in a fluorescence spectrophotometer plate reader, in which peptides are added to bacterial suspensions.

Cell membranes of viable bacteria are polarized (i.e., they have a negative transmembrane potential), and some cationic AMPs kill bacteria by depolarizing their membranes (10, 54). The dye 3,3′-Dipropylthiadicarbocyanine iodide [DiSC_3_(5)] is cationic, membrane-permeable, fluorescent in aqueous environment and can be used to examine the ability of specific AMPs to depolarize bacterial membranes (53, 55, 56). DiSC3(5) has a stable and low fluorescence emission signal when bound to viable bacteria with polarized membranes; if an AMP induces membrane depolarization, the dye is released, and its fluorescence emission intensity increases (Figure 1D). In this assay, it is important to optimize cell density, dye concentration and ensure that tested AMPs do not quench the fluorescence of the dye, as this assay is based on fluorescence quenching (57).

## Examining Bacterial Membrane Integrity Using Flow Cytometry

Flow cytometry combines fluidic, optical and electronic parameters to analyze physical properties (e.g., size and granularity) and fluorescence of individual cells within a population of cells. Each cell goes through a set of lasers and produces scattered and fluorescent light signals that are detected and analyzed by a computer (58).

Bacteria with permeabilized membranes can be distinguished from viable bacterial cells using two fluorescent dyes [e.g., propidium iodide (PI) and SYTO 9] and by analyzing cells using flow cytometry (59). PI and SYTO 9 become fluorescent when intercalating with DNA. However, PI is a red-fluorescent dye, non-permeable to intact plasma membranes and cannot enter viable cells, whereas SYTO 9 is a green-fluorescent dye that can enter both live and dead bacterial cells (Figure 1E). PI has stronger affinity for nucleic acids than SYTO 9; therefore, when both dyes have access to nucleic acids inside bacteria, PI displaces SYTO 9 (60).

There are some factors that might interfere with correct readings, such as photobleaching of SYTO 9, variable binding affinities of SYTO 9 to live and dead cells, background fluorescence and bleed-through (61). Moreover, some bacteria strains have efflux pumps that can remove PI from the cell (60). Nevertheless, this is a rapid and high-throughput assay to quantify the effect of AMPs on the cell membrane integrity within a large population of cells (62).

SYTOX Green is another high-affinity nucleic acid stain that can be used to investigate cell membrane integrity in bacteria using flow cytometry. This dye only enters cells with compromised plasma membrane (Figure 1F). The binding of SYTOX green to nucleic acids results in >500-fold increase in fluorescence emission intensity (63). SYTOX green and PI have similar molecular weight (i.e., 600 and 688 Da), and entry of these two dyes into bacteria is unlikely to be discriminated based on their size. Nevertheless, the detection of permeabilized cells and distinction from non-permeabilized cells is superior when using SYTOX Green. This dye has a higher quantum yield and molar extinction coefficient compared to that of PI (63). These advantages might explain why many studies used SYTOX green to investigate bacterial membrane integrity in the presence of AMPs, instead of the combination of SYTO 9 and PI (53, 64–69).

## Confirming Whether Membrane Disruption is the Cause of Death

It is important to investigate whether peptide concentrations required to lyse bacterial membranes correlate with concentrations required to kill bacteria. Some AMPs display a minimal inhibitory concentration (MIC) below the concentration required to disrupt membranes, suggesting that bacteria are being killed/inactivated by a mechanism not directly related to cell membrane disruption. This can be investigated by treating bacteria with various concentrations of peptide and quantifying viable cells (e.g., using plate colony count method) in parallel with permeabilized cells (e.g., using a flow cytometry assay with SYTOX Green) (67, 70). Interestingly, some bacteria species seem to be more resistant to membrane damage, as suggested by a study with the AMP maculatin 1.1. This peptide induces uptake of SYTOX Green in *E. coli* and *Staphylococcus aureus* at similar concentrations, but is more potent at inhibiting the growth of *S. aureus* (67).

When AMPs inhibit bacterial growth at non-permeabilizing concentrations, their MOA is likely to involve entry in the cell and ability to interact with an intracellular target; therefore, it is important to investigate whether they can enter inside bacteria. These non-lytic AMPs able to cross bacterial membranes can also be referred to as cell-penetrating peptides (66).

## Distinguishing Internalized and Membrane-Bound Peptide Via Flow Cytometry

Uptake into bacterial cells can be investigated using flow cytometry and peptides labeled with a fluorophore, such as Alexa Fluor® 488, fluorescein isothiocyanate (FITC), bodipy or rhodamine. Care must be taken when choosing the fluorophore, as some can alter peptide physicochemical properties (e.g., increase of overall hydrophobicity), peptide-peptide intermolecular interactions, binding affinity for lipid bilayers, activity, or cellular uptake (71–75).

There are also some challenges in conjugating peptides with fluorescent labels, as the most common strategies involve amide bond ligation with labels derivatized with succinimidyl esters and require a free amine (66, 69). Therefore, the peptide requires an uncapped N-terminal, or a Lys residue within the amino acid sequence if the peptide is backbone-cyclised. Moreover, when the peptide has more than one Lys, several isomers with one or more label molecules might be obtained (76). To decrease the number of possible isomers and avoid changes in the overall charge of Lys-rich peptides after labeling, the dye can be conjugated using site-directed strategies. Some examples include conjugation of alkyne-derivatized dyes onto azide-containing peptides with copper-catalyzed azide-alkyne cycloaddition, and conjugation of dyes containing hydroxylamine with acetone-linked peptides using oxime ligation (77).

Fluorescently labeled AMP molecules located inside bacteria can be distinguished from those bound to the bacteria surface by screening peptide-treated cells via flow cytometry (Figure 1G). Fluorescence emission intensity and percentage of fluorescent bacteria are recorded before and after addition of trypan blue (TB), an aqueous fluorescence quencher unable to enter cells with intact membranes. A similar fluorescence emission signal before and after addition of TB indicates that the peptide is inside the bacteria. A decrease in fluorescence emission signal suggests that a proportion of peptide molecules is surface exposed and accessible to TB. A significant decrease in the percentage of fluorescent bacteria and in the fluorescence emission intensity indicate that cells are permeabilized, which enabled TB to enter and quench the fluorescence emission of peptide inside cells (66, 78, 79).

## Using Microscopy to Visualize Bacteria Morphology and Peptide Location

Changes in cell morphology induced by AMPs can be visualized using electron microscopy and atomic force microscopy (AFM). Bacterial cells incubated with sub-lethal and/or lethal concentrations of AMPs are imaged to identify whether the membrane surface is intact, becomes wrinkly, has blebs, or is lysed (28, 80–82). AMPs inducing visible damages are likely to act on the membrane, whereas lack of morphological changes at lethal concentrations suggests interference with an internal component.

The location of labeled AMPs inside bacteria can be visualized using confocal microscopy. For instance, AMPs NCR247 and NCR235 bind to the membrane of both *Salmonella* and *Listeria*, but can only reach the cytosol of *Salmonella* (83, 84). In another study, the authors showed localization of an AMP inside *E. coli* using confocal microscopy, SYTOX green and rhodamine-labeled AMP, and confirmed changes in the *E. coli* morphology using AFM (70). These examples demonstrate how diverse and versatile microscopy techniques are to evaluate peptide location within bacteria, and effect on their membrane.

## Detection of Amp and Nucleic Acid Interactions Using Electrophoretic Mobility Shift Assays

Some AMPs kill bacteria by interacting with DNA or RNA, and therefore interfere with their synthesis, replication, and translational processes (10, 85). It is debatable whether AMPs can target specific portions of DNA/RNA, as positively charged AMPs might bind unspecifically to negatively charged nucleic acids via strong electrostatic attractions (86). In addition, binding to nucleic acids can be unrelated to the cell death mechanism, as it can occur as a result of AMPs entering bacteria after disrupting their membranes. Thus, studies to investigate interaction with DNA/RNA are more appropriate with non-lytic AMPs.

**Figure 2:**
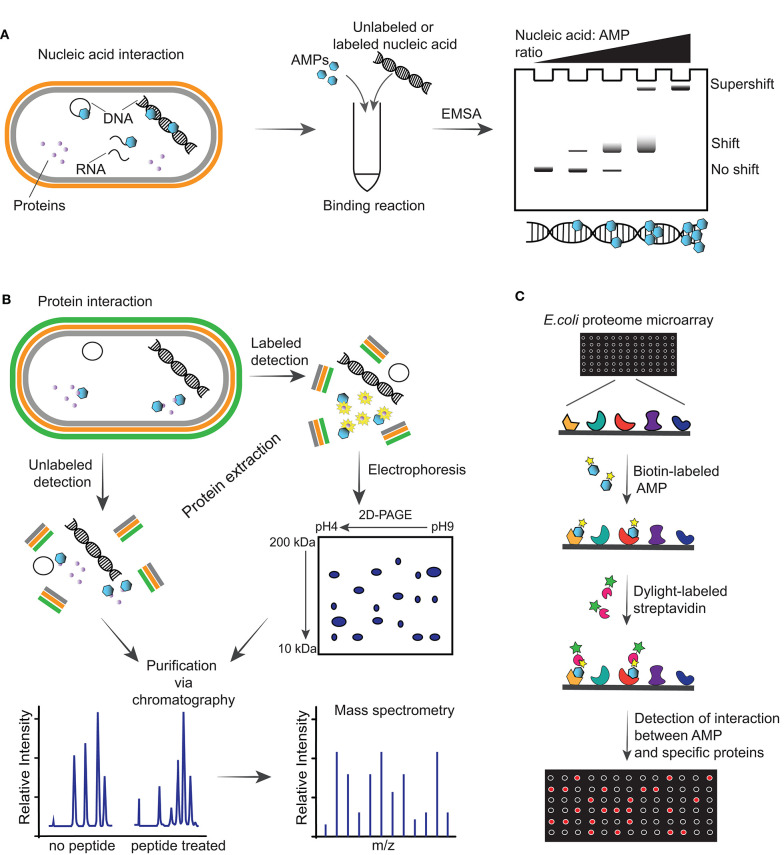
Illustrations of methodologies for detection of intracellular targets of antimicrobial peptides (AMPs). **(A)** Schematic of detection of an AMP able to interact with nucleic acid including DNA, plasmid DNA and RNA of a Gram-negative bacterium via electrophoretic mobility shift assay (EMSA). This assay consists of running incubation reactions between increasing ratios of AMPs with specific nucleic acid (labeled or unlabeled) and running the reactions through an agarose gel. Depending on the affinity of the AMP with the nucleic acid, a shift or supershift in the EMSA is observed. **(B)** Interaction of AMPs with intracellular proteins can be detected by incubating the peptides with bacteria, followed by protein extraction, purification, and mass spectrometry analysis. Alternatively, cells can be incubated with AMPs followed by addition of radioactive labels to incorporate labels to the newly synthesized proteins and performing a two-dimensional polyacrylamide gel (2D-PAGE), from which spots are analyzed via mass spectrometry. **(C)** Schematic representation of *E. coli* proteome microarray and workflow. Biotinylated AMPs are probed on the microarray followed by Dylight-labeled streptavidin to tag the biotin linked on the AMPs and signals are obtained by scanning the chip with a laser scanner to show interaction between AMPs and specific proteins on the chip.

The gel electrophoretic mobility shift assay (EMSA) is a rapid and sensitive methodology used to detect interactions between proteins, or peptides, with DNA/RNA (87). In this assay, the peptide is incubated with nucleic acids and the reaction is electrophoresed on an agarose or native polyacrylamide gel to detect whether a peptide-nucleic acid complex is formed based on a slower migration than that of free nucleic acid. The complex can be detected using a range of approaches, including fluorescence, chemiluminescence, immunohistochemical and highly sensitive radioisotope-labeled nucleic acids (Figure 2A). One limitation to consider during the electrophoresis is that samples are not at chemical equilibrium and therefore rapid dissociation during this time might prevent detection of the complex. Antimicrobial peptides such as indolicin (88), LL-37 (89), Burforin II (90) and Frenatin 2.3S peptide (91) have been shown to bind bacterial DNA using EMSAs.

## Proteomics to Search For a Putative Intracellular Target Or/and Differences in Protein Expression

Non-lytic AMPs that translocate through bacterial membranes might inhibit intracellular processes by interacting with essential proteins and enzymes (92). Mass spectrometry and proteome microarray are examples of methodologies used to identify protein targets of AMPs and/or the subsequent changes in protein expression due to peptide entry inside bacteria.

Mass spectrometry is a powerful tool to examine changes in protein expression of bacteria treated with AMPs. In the unlabeled approach, after co-incubation of bacteria with AMPs, proteins are extracted, digested and fractionated, and the whole proteome is analyzed by mass spectrometry (Figure 2B). A recent study used this approach to show the effect of LL-37 peptide on the proteome of *Streptococcus pneumoniae* D39, and identified alteration in the expression of 105 proteins (93). Treatment with LL-37 induced upregulation of proteins involved in cell surface modification, including increasing membrane surface charge, and an abundance of ABC transporters. These modifications are likely to help removing LL-37 from the bacterial membrane. Using a similar workflow, lipidomics studies can be used to investigate changes in the lipid composition of bacterial membranes after treatment with AMPs, as recently reviewed (94).

Wenzel et al. (95) reported a radio-labeling approach to facilitate the identification of proteins that are up/down regulated upon treatment with peptide (Figure 2B). In this study, *Bacillus subtilis* cultures were incubated with RW-rich peptides, followed by addition of radioactive methionine to incorporate a radioactive label into newly synthesized proteins. Proteins were extracted from cells and separated on a two-dimensional polyacrylamide gel (2D-PAGE) where up/down regulated proteins were identified, excised from the gel and identified by mass spectrometry (95).

Proteome microarray is another powerful and high-throughput tool with potential to screen the entire proteome and identify targets in a single experiment. This has been exemplified with a biotin-labeled AMP and incubated with an *E. coli* K12 proteome microarray chip, followed by detection of the biotinylated AMPs with Dylight-labeled streptavidin (92) (Figure 2C). The signals detected on a microarray scanner show binding between specific proteins and AMPs. Fabricated *E. coli* K12 proteome microarrays have been used to identify intracellular protein targets of several AMPs, such as bactenecin 7, a hybrid of pleurocidin and dermaseptin, and proline-arginine-rich peptide (96). However, the coverage of the microarray, differences between the proteins immobilized on microarrays and their counterpart in physiological conditions, and the cost are limitations for a broader use of protein microarrays (97).

## Discussion

AMPs are promising leads to develop new antimicrobial drugs to treat bacterial infections. The number of AMPs reported to date is high, and some candidates reached clinical trials (98, 99). Understanding the MOA used by AMPs to kill bacteria is important to advance their development. Here we describe some experimental biophysical tools that can be used to investigate whether AMPs act by disrupting bacterial membranes, or by modulating intracellular activities. An overview of multiple AMPs, their MOA and the biophysical techniques employed to characterize them, is summarized in a recent review (100).

Determining whether an AMP disrupts bacterial membranes can be straightforward; however, finding specific intracellular targets is more complex. Pinpointing whether AMPs act by interfering with DNA, RNA or proteins remains challenging, as some AMPs not only act on the membrane they can also activate a cascade of reactions within the bacteria (12, 85). Advances in transcriptomics, proteomics, lipidomics and continued development of high-throughput techniques will facilitate these MOA studies.

Computational tools, such as molecular dynamics simulations, machine learning, and AMPs databases [such as https://dbaasp.org/ (101)] are also important to identify new AMPs, characterize their MOA, and predict lead candidates (102, 103). Computer-based and wet-lab based methodologies can be integrated to better understand the MOA of AMPs, and to rationally design novel AMPs with a required MOA. For example, when searching for AMPs to target intracellular pathogens, it is desirable to have peptides with cell-penetrating properties able to enter host cells and kill bacteria by modulating specific bacterial components, without disrupting the membrane of host cells. On the other hand, when targeting mixed-species biofilms, membrane-lytic AMPs with a broad-spectrum activity are likely to be more effective at the infection site, compared to AMPs that selectively inhibit an intracellular target.

MOA studies *in vitro* and *in silico*, together with efficacy, safety and pharmacology studies *in vivo* and *ex vivo*, are essential to convert AMP leads into therapeutics used in the clinic. In particular, MOA studies help in identifying off-targets in host cells, understanding spectrum of activity, finding intrinsically resistant bacteria, and overcoming challenges associated with delivery of peptides. Therefore, the biophysical tools here described can assist the development of novel antibiotics.

## Author Contributions

AB and STH conceptualized the manuscript. AB wrote the original draft. STH reviewed and edited the manuscript. Both authors contributed to the article and approved the submitted version.

## Conflict of Interest

The authors declare that the research was conducted in the absence of any commercial or financial relationships that could be construed as a potential conflict of interest.
